# Gender Incongruence of Childhood: Clinical Utility and Stakeholder Agreement with the World Health Organization’s Proposed ICD-11 Criteria

**DOI:** 10.1371/journal.pone.0168522

**Published:** 2017-01-12

**Authors:** Titia F. Beek, Peggy T. Cohen-Kettenis, Walter P. Bouman, Annelou L. C. de Vries, Thomas D. Steensma, Gemma L. Witcomb, Jon Arcelus, Christina Richards, Griet De Cuypere, Baudewijntje P. C. Kreukels

**Affiliations:** 1 Center of Expertise on Gender Dysphoria, VU University Medical Center, Amsterdam, The Netherlands; 2 Department of Medical Psychology, VU University Medical Center, Amsterdam, The Netherlands; 3 Nottingham Centre for Gender Dysphoria, Nottingham, United Kingdom; 4 Department of Child and Adolescent Psychiatry, VU University Medical Center, Amsterdam, The Netherlands; 5 School of Sport, Exercise, and Health Sciences, Loughborough University, Loughborough, United Kingdom; 6 Division of Psychiatry and Applied Psychology, Faculty of Medicine & Health Sciences, University of Nottingham, Nottingham, United Kingdom; 7 Center for Sexology and Gender, Ghent University Hospital, Ghent, Belgium; University of Toronto Dalla Lana School of Public Health, CANADA

## Abstract

The World Health Organization (WHO) is revising the tenth version of the International Classification of Diseases and Related Health Problems (ICD-10). This includes a reconceptualization of the definition and positioning of Gender Incongruence of Childhood (GIC). This study aimed to: 1) collect the views of transgender individuals and professionals regarding the retention of the diagnosis; 2) see if the proposed GIC criteria were acceptable to transgender individuals and health care providers; 3) compare results between two countries with two different healthcare systems to see if these differences influence opinions regarding the GIC diagnosis; and 4) determine whether healthcare providers from high-income countries feel that the proposed criteria are clinically useful and easy to use. A total of 628 participants were included in the study: 284 from the Netherlands (NL; 45.2%), 8 from Flanders (Belgium; 1.3%), and 336 (53.5%) from the United Kingdom (UK). Most participants were transgender people (or their partners/relatives; TG) (n = 522), 89 participants were healthcare providers (HCPs) and 17 were both HCP and TG individuals. Participants completed an online survey developed for this study. Overall, the majority response from transgender participants (42.9%) was that if the diagnosis would be removed from the mental health chapter it should also be removed from the ICD-11 completely, while 33.6% thought it should remain in the ICD-11. Participants were generally satisfied with other aspects of the proposed ICD-11 GIC diagnosis: most TG participants (58.4%) thought the term Gender Identity Disorder should change, and most thought Gender Incongruence was an improvement (63.0%). Furthermore, most participants (76.1%) did not consider GIC to be a psychiatric disorder and placement in a separate chapter dealing with Gender and Sexual Health (the majority response in the NL and selected by 37.5% of the TG participants overall) or as a Z-code (the majority response in the UK and selected by 26.7% of the TG participants overall) would be preferable. In the UK, the majority response (35.8%) was that narrowing the GIC diagnosis was an improvement, while the NL majority response (49.5%) was that this was not an improvement. Although generally the results from HCPs were in line with the results from TG participants some differences were found. This study suggests that, although in an ideal world a diagnosis is not welcomed, several participants felt the diagnosis should not be removed. This is likely due to concerns about restricting access to reimbursed healthcare. The choice for positioning of a diagnosis of GIC within the ICD-11 was as a separate chapter dealing with symptoms and/or disorders regarding sexual and gender health. This was the overall first choice for NL participants and second choice for UK participants, after the use of a Z-code. The difference reflects that in the UK, Z-codes carry no negative implications for reimbursement of treatment costs. These findings highlight the challenges faced by the WHO in their attempt to integrate research findings from different countries, with different cultures and healthcare systems in their quest to create a manual that is globally applicable.

## Introduction

The World Health Organization (WHO) is currently revising the tenth version of the International Classification of Diseases and Related Health Problems (ICD-10; [1]). This includes a reconceptualization of the definition and positioning of the current ICD categories of gender identity related diagnoses. In many health care systems a diagnosis is required for people to access the care, including gender-affirming medical treatment [2]. However, including transgender people as part of a diagnostic category can [psycho]pathologize and stigmatize transgender individuals—particularly if these categories remain within the chapter on Mental and Behavioural Disorders as in the ICD-10. The WHO received a large number of calls to remove transgender diagnoses from the mental disorders section of the classification system (e.g. of Global Action for Trans* Equality (GATE) [3] and the International Campaign Stop Trans Pathologization (STP) [4]). The European Parliament also called upon the Commission and the World Health Organization to withdraw gender identity diagnoses from the mental and behavioural disorders chapter, and to ensure a non-pathologizing reclassification in the ICD-11 [5].

One possible solution that could help to reduce stigma and pathologization of transgender people would be to include the Gender Incongruence diagnoses, Gender Incongruence of Adolescence or Adulthood (GIAA) and Gender Incongruence of Childhood (GIC) as Z-codes (see, for example [3,6]). Z-codes concern “factors influencing health status and contact with health services” (ICD-10), but do not concern diseases or disorders. Z-codes are used in situations where something other than a disease, injury or external cause (e.g., poisoning) requires contact with medical organizations. These are then mentioned as the 'diagnosis' or 'problem’. Examples of Z codes include problems related to certain psychological circumstances, such as unwanted pregnancy; counselling related to sexual attitude, behavior, and orientation; and psychosocial conditions or problems related to lifestyle or life-management.

One of the proposals for the new ICD is to change the current diagnostic term of *Transsexualism* to *Gender Incongruence* (GI) (see [2]). GI would then be included as a Z-code and, rather than being seen as a disorder or a disease, it would be seen as something that might affect health. This could help reduce stigma among transgender people, but it may also have disadvantages. For example, in some countries, such as the Netherlands, Z-code conditions are not reimbursed or covered by health insurers—which would leave many transgender people without access to clinical services. In other countries, such as the United Kingdom, a change to Z-codes would have no impact on payment for services and their consequent provision. Another debate related to the new classification system revolves around the diagnosis of Gender Incongruence of Childhood. While some people propose the removal of this diagnosis from the classification system, others feel that being part of it is important. Proponents for the removal of this diagnosis state that a diagnosis for children is not needed because: 1) no medical treatment for pre-pubertal children with gender incongruence of childhood is available (e.g., [3,6]); 2) it stigmatizes and pathologizes children with normal variations of gender expressions [3,6]; 3) due to the fact that it is not possible to predict which children will have persisting gender incongruence of adolescence or adulthood and which will not [7–10], clinical input will be provided to children when it is not needed thus increasing the risk of iatrogenic harm [6]; and 4) the way gender variance in children is viewed is culture-specific, with many non-Western cultures being more accepting of gender variance than Western cultures [6].

Proponents for the retention of this diagnosis for children [6] argue that it is required because: 1) parents will benefit from the help, support and advice associated with it; 2) in many healthcare systems a diagnosis is needed to access reimbursed care and (specialized) information; 3) it provides legal protection and a “protected status” (i.e., non-discrimination, accommodations in school); 4) it facilitates professional training and expertise; and 5) it facilitates research to improve the quality of care for children and their families.

In 2013, the World Professional Association for Transgender Health (WPATH) held an expert consensus meeting to discuss whether the diagnostic category of Gender Incongruence of Childhood should be removed from the ICD 11 or retained. Consensus was not reached as votes were equally split with fourteen members voting for removal and fourteen voting for retention of a diagnosis for children with gender incongruence [6]. A recent survey among WPATH members concerning the proposed ICD-11 Gender Incongruence of Childhood diagnosis has had similar results [11].

### Proposed changes to the GIC classification

The above mentioned dilemma—regarding how best to provide a classification that ensures access to care while at the same time providing recognition rather than pathologization or stigmatization—provided the background to the current proposal of the WHO’s Working Group on the Classification of Sexual Disorders and Sexual Health (WGSDSH). Several recommendations relevant to the GIC diagnosis were made by this group (for proposed GIC criteria see [12]):

1. The diagnosis would no longer be included in the mental health chapter, but be part of an independent chapter on sexual health.
2. The ICD diagnostic term to be used would be changed from *Gender Identity Disorder of Childhood* into *Gender Incongruence of Childhood*.
3. Stricter criteria for the new diagnosis of GIC would be used in order to reduce the risk of false positives.
4. Distress due to gender incongruence would not form part of the diagnostic criteria.

The working group noted:

“…there are individuals who today present for gender reassignment who may be neither distressed nor impaired. This may be particularly true for young adolescents who are aware of the possibility of gender transition, live in an accepting environment, and who can have access to puberty suppressing treatments until they are able to take such a decision.” [2]

The WHO published a beta draft of the proposed ICD-11 in order to receive input on the proposed structure and diagnostic criteria (see [12]). As indicated above, Gender Incongruence of Adolescence and Adulthood and Gender Incongruence of Childhood are placed in the proposed new chapter entitled ‘Conditions related to sexual health’, which is a deviation from the preferences for placement of the WGSDSH, whose first preference was for placement in a separate chapter focused on gender incongruence only [2]. Their second preference was for the gender incongruence diagnoses to be placed in a new chapter focusing on sexual and gender health [2]. In the current ICD beta version, the focus on gender is lost and reads: Conditions related to Sexual Health, see [12]. The suggested changes concerning placement and characteristics of the childhood diagnosis were made by the WGSDSH based on the assumption that the diagnosis would remain part of the ICD-11.

### Field testing

The WHO has subjected the WGSDSH’s initial recommendations to field testing in a variety of relevant health care settings in different WHO regions. In all previous revisions, Gender Incongruence has never been subject to formal field-testing. The WHO has decided that the new revision should improve clinical utility. WHO views clinical utility as a global public health issue [13]. Indeed, clinicians consider an important purpose of a diagnostic system to be a facilitator of communication among clinicians and to inform treatment and management. Rather than a strict criteria-based approach, clinicians prefer a simple and descriptive system that can be used by specialists as well as general healthcare providers, in high- as well as middle- and low-income countries [14].

The WHO is already performing field studies in several low- and middle-income countries regarding these issues. However, field studies are also necessary in countries with well-developed healthcare systems, since some of the controversial issues can only be tested in clinical settings where a sufficient number of patients are assessed. This is particularly the case for children and adolescents with gender incongruence, as there are few clinical centres in the world that are accessible to those children. These are the reasons why the Netherlands and the United Kingdom (UK) have been selected to undertake these field studies.

### The Dutch and UK healthcare systems

Healthcare systems differ across the world, and also between the Netherlands and the United Kingdom. As the way healthcare is organized might influence ideas about diagnostic classification, a short overview of the Dutch and UK systems and the organization of transgender healthcare is provided. It is mandatory for people who legally live or work in the Netherlands to have basic healthcare insurance and several insurance providers exist (some for-profit, others non-profit) that allow consumers choice during annual open enrolment periods. These companies are obliged to accept every person for the basic package and are not allowed to select only people with low health risks (e.g., the young, not having a chronic illness). Discrimination based on health risks and conditions is therefore not allowed [15]. Consumers may, however, choose to buy supplemental insurance covering care that is not included in the mandatory basic insurance. For this supplementary health insurance, insurers are not required to accept all applicants [16].

Applicants for transgender related healthcare within this system are referred through a general practitioner and healthcare reimbursement is based on their diagnosis or that of another appropriate professional. Regarding specialist care in the Netherlands, multidisciplinary transgender related healthcare can be obtained at three centres: Leiden University Medical Centre (children and adolescents), University Medical Centre Groningen (adults), and the Center of Expertise on Gender Dysphoria (CEGD) of the VU University Medical Centre in Amsterdam (children, adolescents, and adults). A number of other hospitals do not have multidisciplinary teams, but do provide transgender related surgery after referral. Aside from this, some transgender people go abroad to obtain surgical interventions, though this is not always reimbursed by healthcare insurers.

The United Kingdom is made up of four countries (England, Scotland, Wales, and Northern Ireland) each of which has a separate National Health Service (NHS) and all of whom use the ICD-10 [1]. The NHS is funded through central taxation and provides a comprehensive range of health services, the vast majority of which are free at the point of access for people legally resident in the United Kingdom. It is not an insurance-based system and although ICD diagnoses are collected, the funding is not dependent upon them. Therefore diagnoses which are part of Z-codes are normally funded. Services for adult people (>17 yrs) with gender dysphoria who live in England and Wales are funded by NHS England and are provided via seven NHS Gender Identity Clinic services, all of which are based in England. There is also one Gender Identity Clinic service in Glasgow, Scotland, and one in Belfast, Northern Ireland. The clinical services were originally developed according to the interests of individual clinicians, rather than on a national, strategic level, hence the lack of these services in some parts of country such as the North-West. As in the Netherlands, applicants of transgender related healthcare are referred through a general practitioner, although clinicians in secondary care services can also refer. Currently, there is only one NHS service for children and adolescents with gender dysphoria in the United Kingdom which is based in London, with two satellite clinics in Exeter and Leeds, England.

### Aim of the study

This field study aimed to: 1) collect the views of transgender individuals and professionals regarding the retention of the diagnosis; 2) see if the proposed GIC criteria were acceptable to transgender individuals and health care providers 3) compare results between two countries with two different healthcare systems to see if these differences influence opinions regarding the GIC diagnosis; and 4) determine whether healthcare providers from high-income countries feel that the proposed criteria are clinically useful and easy to use.

## Methods and Materials

Methodology, sample characteristics, and procedure have been described previously [17], since data on both the GIC and GIAA diagnosis were collected in one survey.

### Methods

#### Participants & procedure in the Netherlands and Flanders, Belgium (NL)

Participants in this study consisted of healthcare providers as well as consumers of transgender healthcare and stakeholders (i.e., parents or siblings of transgender children, or partners of transgender persons). Only people over the age of 16 years were included. Due to the complexity of the questions, the questionnaire was not suitable for children. Mental health professionals specialized in gender incongruence were recruited from the VUmc Center of Expertise on Gender Dysphoria (CEGD) in Amsterdam, the Netherlands; the Department of Sexology and Gender of the University Hospital in Ghent in Flanders, Belgium; the Gender team of the University Medical Center Groningen, the Netherlands; and Transvisie Zorg Amsterdam, the Netherlands—an organization that provides information, counselling and psychological care for transgender people and those in their social environment. Specialized healthcare providers were sent a link to an online survey to complete. As one of the aims of the WHO is to provide criteria that are easy to use for health care providers across the world, healthcare providers who were *not* specialized in transgender care (e.g., healthcare psychologists, psychiatrists, general practitioners, general practitioners trainees, social workers) were recruited to take part as well. They were contacted via e-mail through the researchers’ networks and after agreeing to participate were invited via e-mail to the online survey. Furthermore, general psychiatrists and psychiatric trainees were invited to participate in the survey following a research session organized by the Dutch researchers. Some participants completed the online version, while others completed a pen and paper version of the survey. For the recruitment of service users, transgender adults who came to the Vumc Amsterdam Center of Expertise on Gender Dysphoria (CEGD) for an appointment were asked if they would like to participate in the study. Parents of transgender children were approached through a mailing of Berdache, a Dutch support group for parents of transgender children. In addition, parents were approached when they came to the CEGD at the Vumc. The researchers sent out personal survey links to those interested in participating.

A total of 758 people were invited to participate in the Dutch survey (from the Netherlands and Belgium). Of this group, 36 people refused participation for various reasons (multiple response options were possible): they did not feel like participating (*n* = 16); they did not have time (*n* = 11); it did not matter to them whether or not the diagnosis (terminology and criteria) was changed (*n* = 8); and other reasons (*n* = 5). Ultimately, 722 agreed to participate and received a link to the survey via e-mail. Despite several reminders, 383 people (53.0%) failed to open the link to start the survey. At the start of the survey, participants first received information about the study and were then asked to give written consent by selecting “yes” in response to the question: “Do you agree to participate in this study?”. The study was targeted towards participants who were 16 years or older. No consent was collected from parents or caretakers for those aged 16 as these younger participants could give consent themselves (in both countries, the ethics committees agreed with this approach). Forty-one people gave consent but did not answer any questions so they were removed from the data set. Another 6 participants were excluded as they answered fewer than 5 questions. A total of 292 Dutch-speaking participants (284 from the Netherlands, 8 from Belgium) started the survey and answered at least 5 questions (40.4% of those who received a link to the survey and 38.5% of all participants who were approached). All participants from Belgium were specialized healthcare providers. The survey was not presented to transgender people/or their partners or relatives in Belgium. The survey was completed by 223 people (76.4% of included Dutch participants).

#### Participants & procedure in the United Kingdom (UK)

Researchers at the Nottingham Centre for Gender Dysphoria (NCGD) contacted 8 Gender Identity Clinic Services in the United Kingdom and four surgical Centres specialized in transgender surgery, to inform them of the study and ask for assistance in recruitment and participation. The clinicians at the clinics, including the NCGD, were asked to hand the flyers to their patients and, if possible, give a brief explanation of the study. Patients were encouraged to share the online link with friends and family, and clinicians were encouraged to share it with their colleagues within gender and non-gender specialist services, and it was expected that additional participants would be recruited via such snowballing methods.

In the UK sample, due to the snowballing method, the number of invitations sent out is unknown. Overall, 552 UK participants entered the survey. In total, 387 completed the survey to the end (70.1%) and only their responses were saved by the survey software. Seven participants did not progress past the information page, but all others continued on to give consent. Participants then dropped out at various stages, the greatest drop-outs (*n* = 71) occurring for questions regarding the positioning of the diagnosis. Of the 387 participants who completed the survey in full, 50 were not from the United Kingdom and were excluded. One other person was excluded because he/she was under the age of 16. This resulted in a final UK sample of 336 participants.

#### Participants overall

A total of 628 participants were included in data analysis: 292 from the Netherlands (*n* = 284) and Flanders, Belgium (*n* = 8); and 336 (53.5%) from the UK.

### Materials

The research team from the Center of Expertise on Gender Dysphoria of the VU University Medical Center Amsterdam, developed a questionnaire with the input of experts and stakeholders [17]. This questionnaire covered questions regarding both the Gender Incongruence of Childhood classification (GIC) and the Gender Incongruence of Adolescence and Adulthood (GIAA). The results of the survey questions regarding the GIAA diagnosis are reported elsewhere, see [17]. The final survey consisted of two parts (see S1 Text for the complete survey). The first part focused on the view of the service users and clinicians with regard to the various changes proposed in the ICD-11 (as compared to the ICD-10). A special effort was made to explain concepts that some participants may have been unfamiliar with through the use of pop-up information windows accessible via a mouse click on blue-colored, bold and underlined words. For example, explanations were provided on the concept of a Z-code (this concerns “factors that affect health and also influence contacts with the healthcare system” but does not concern diseases or disorders). The second part of the survey was available for healthcare providers only (including transgender participants who were healthcare providers) and aimed to examine the clinical utility and the clinical implications of the proposed GIC diagnosis. The ICD-11 GIC criteria used were the final draft criteria of the WGSDSH (see S2 Text).

The Dutch survey went live on June 2^nd^ 2014 and was open for 10.5 months. For the UK study, the Dutch version of the questionnaire was translated into English by a Dutch translation company and then further amendments to the language were made to ensure appropriateness and ease of use by lay people. The questionnaire was then sent to the lead clinicians at all Gender Identity Clinic services in the United Kingdom, as well as the main providers of gender-related surgery. A meeting took place where stakeholders could comment on the questionnaire and no major amendments were suggested, but the length of the questionnaire was questioned by various stakeholders. However, changes to this could not be made for reasons of consistency with the original Dutch version. During the same period, representatives from the major trans stakeholder groups in the United Kingdom were invited to meet and discuss the questionnaire in detail. The overarching remit given to this group was to remain as closely as possible to the original Dutch version of the questionnaire. Amendments were made to some of the language used, the order of questions, response options, and a small number of new questions was added. The final version was then translated back into Dutch by another Dutch translation company and shared with the team in the Netherlands. The online questionnaire went live in the UK on October 1^st^ 2014 and was open for 7.5 months (see also [17]).

The survey began with a series of demographic questions that asked about age, geographical location, gender identity (identification and description), birth assigned gender, education, employment status, and respondent category (i.e., trans, relative, healthcare provider). It then went on to ask about general opinions towards diagnosis and whether the respondent was receiving or had received treatment (if applicable). All questions were fixed-choice options, with spaces provided to answer “other” and add more information if required. The answer options going through the survey were a mixture of one-option responses or multi-option responses, depending upon the question. Options were provided to cover all eventualities, including neutral/no opinion; always with the addition of “other, please specify” or “please explain your answer” to allow for additional comments. For example, questions that asked for level of agreement would give seven main options: “Strongly agree, Agree, Agree a little, Neutral; Neither agree nor disagree, Disagree a little, Disagree, and Strongly disagree”, plus “please explain your answer”. Questions that asked about opinions (e.g., “Do you think….”) would give four main options: “Yes, No, No opinion, Don’t know” plus “please explain your answer” (see also [17]).

The Dutch study was granted full ethical approval (inclusion of Belgian participants and the consent method for participants under the age of 18 was covered) by the Commissie Wetenschappelijk Onderzoek [Committee of Scientific Research] of the EMGO Institute for Health and Care Research (EMGO^+^) with project ID WC2014-09. The UK study was granted full ethics approval (the consent method for participants under the age of 18 was covered) by the UK National Research Ethics Service Committee East Midlands—Nottingham 1 with IRAS project ID 152591.

### Data analysis

Participants who were both healthcare providers and transgender were included in both the Transgender group (TG) and the Healthcare providers (HCP) group. Differences between participants from the United Kingdom (UK) and participants from the Netherlands/Flanders (NL) were explored with a t-test for continuous data (age), Chi-square tests or Fisher’s Exact test (for the HCP only questions where the sample was smaller and Chi-square assumptions were violated) for categorical data and Mann-Whitney U tests for ordinal data or when t-test assumptions were violated. As participants who were both HCP and TG persons were included in both groups, the TG and HCP group were not independent. Therefore, no statistical differences between these groups were explored. The results of the HCP group are described after the TG group and finally, the findings from the specific questions for healthcare providers are described.

## Results

### Sample characteristics

Based on the participant type categories selected (see S3 Text for the additional information on respondent categories), participants were divided into three categories: healthcare providers (HCP); service users (transgender persons and their partners and/or relatives; TG); and participants who are both healthcare providers and (partners/relatives of) transgender persons. The TG group consists mainly of transgender persons (see Table A in S3 Text), but also partners, relatives, and parents of children/adolescents with gender incongruent feelings were included, as they have first-hand experience with the healthcare provided for their family member. Most participants were in the TG group (*n* = 522), 89 participants were HCP, and 17 participants were both HCP and TG (see Table 1). These three categories were used to analyse the responses regarding the survey questions. Note that when we refer to the participants from the Netherlands and Flanders, the abbreviation NL is used.

**Table 1:** Frequency Table of Gender Assigned at Birth, Gender Identity, and Level of Education (and Percentages for each Column). Table copied from [17].

Demographic			Respondent Category										
			All		(Relatives/Partners of) transgender people			Healthcare providers			Both healthcare providers and (relatives/partners of) transgender people		
			Country of data collection		Country of data collection			Country of data collection			Country of data collection		
		Total	NL	UK	NL	UK	Total	NL	UK	Total	NL	UK	Total
		(*n* = 628)	(*n* = 292)	(*n* = 336)	(*n* = 210)	(*n* = 312)	(*n* = 522)	(*n* = 72)	(*n* = 17)	(*n* = 89)	(*n* = 10)	(*n* = 7)	(*n* = 17)
Assigned gender	Male	334 (53.2%)	123 (42.1%)	211 (62.8%)	103 (49.0%)	195 (62.5%)	298 (57.1%)	17 (23.6%)	13 (76.5%)	30 (33.7%)	3 (30.0%)	3 (42.9%)	6 (35.3%)
	Female	292 (46.5%)	169 (57.9%)	123 (36.6%)	107 (51.0%)	115 (36.9%)	222 (42.5%)	55 (76.4%)	4 (23.5%)	59 (66.3%)	7 (70.0%)	4 (57.1%)	11 (64.7%)
	Neither	2 (0.3%)	0 (0.0%)	2 (0.6%)	0 (0%)	2 (0.6%)	2 (0.4%)	0 (0%)	0 (0%)	0 (0%)	0 (0%)	0 (0%)	0 (0%)
Gender Identity	Male	187 (29.8%)	104 (35.6%)	83 (24.7%)	81 (38.6%)	67 (21.5%)	148 (28.4%)	17 (23.6%)	13 (76.5%)	30 (33.7%)	6 (60.0%)	3 (42.9%)	9 (52.9%)
	Female	346 (55.1%)	163 (55.8%)	183 (54.5%)	105 (50.0%)	177 (56.7%)	282 (54.0%)	55 (76.4%)	4 (23.5%)	59 (66.3%)	3 (30.0%)	2 (28.6%)	5 (29.4%)
	Partly male, partly female	27 (4.3%)	10 (3.4%)	17 (5.1%)	9 (4.3%)	17 (5.4%)	26 (5.0%)	0 (0%)	0 (0%)	0 (0%)	1 (10.0%)	0 (0%)	1 (5.9%)
	Neither male nor female	26 (4.1%)	4 (1.4%)	22 (6.5%)	4 (1.9%)	22 (7.1%)	26 (5.0%)	0 (0%)	0 (0%)	0 (0%)	0 (0%)	0 (0%)	0 (0%)
	Don't know (yet)	5 (0.8%)	0 (0.0%)	5 (1.5%)	0 (0%)	5 (1.6%)	5 (1.0%)	0 (0%)	0 (0%)	0 (0%)	0 (0%)	0 (0%)	0 (0%)
	Other	37 (5.9%)	11 (3.8%)	26 (7.7%)	11 (5.2%)	24 (7.7%)	35 (6.7%)	0 (0%)	0 (0%)	0 (0%)	0 (0%)	2 (28.6%)	2 (11.8%)
Level of Education	Low	20 (3.2%)	15 (5.1%)	5 (1.5%)	15 (7.1%)	5 (1.6%)	20 (3.8%)	0 (0.0%)	0 (0.0%)	0 (0.0%)	0 (0.0%)	0 (0.0%)	0 (0.0%)
	Middle	251 (40.0%)	110 (37.7%)	141 (42.0%)	109 (51.9%)	141 (45.2%)	250 (47.9%)	0 (0.0%)	0 (0.0%)	0 (0.0%)	1 (10.0%)	0 (0.0%)	1 (5.9%)
	High	357 (56.8%)	167 (57.2%)	190 (56.5%)	86 (41.0%)	166 (53.2%)	252 (48.3%)	72 (100%)	17 (100%)	89 (100%)	9 (90.0%)	7 (100%)	16 (94.1%)

The age of the participants ranged between 16 years and 78 years with a mean of 38.72 years (*SD* = 14.59). There was no statistical difference between the mean age in the Netherlands (*M* = 38.47, *SD* = 13.64, *n* = 290) and the UK (*M* = 38.94, *SD* = 15.38, *n* = 336), *t* (624) = -.40, *p* = .69). The level of education did not differ between the Netherlands and the UK (*U* = 48589.5, *p* = .81). For sociodemographic characteristics on assigned gender, gender identity, and level of education see Table 1 below.

### General features of the diagnosis

#### Terminology

Respondents were asked what they thought of the statement that the ICD-10 term *gender identity disorder* should change on a seven point scale (from strongly agree (1) to strongly disagree (7)). In the TG group (*n* = 526), the mean agreement was 3.01 (*SD* = 1.71). When comparing the responses between the two countries a statistical difference was found (*U* = 25259.5, *z* = -4.66, *p* < .001, *r* = -.20). The UK TG participants had a lower score (*M* = 2.73, *SD* = 1.63, *n* = 319, mean rank = 239.18) than the NL TG participants (*M* = 3.45, *SD* = 1.76, *n* = 207, mean rank = 300.97), indicating that the UK participants agreed more strongly with the statement than the NL participants. When the possible responses were grouped into three categories (agree to various degrees, neutral, disagree to various degrees), it was found that 64.6% (*n* = 206) of the UK TG participants, agreed with the statement compared to 48.8% (*n* = 101) of the NL TG participants. Of the NL participants, 30.4% (*n* = 63) reported being neutral compared to 23.2% (*n* = 74) of the UK participants. In NL 20.8% (*n* = 43) and in the UK 12.2% (*n* = 39) disagreed with the statement. Like the TG participants, the majority of HCPs (77.7%; *n* = 80) agreed (to various degrees) with the statement that the ICD-10 diagnostic term of *gender identity disorder* should change.

The term *gender incongruence* (GI) was considered an improvement over the term *gender identity disorder* (GID) by 63.0% (*n* = 201) of the transgender participants in the UK sample (this question was not present in the Dutch survey), 20.4% (*n* = 65) did not think the term was an improvement, 10.3% (*n* = 33) said it did not matter and the remaining 6.3% (*n* = 20) had no opinion. Most healthcare providers from the UK (87.5%; *n* = 21) also thought GI was an improvement over GID.

#### Positioning of the Diagnosis

Of all response options available, when asked where should the diagnosis go if removed from the mental health chapter, the most frequently selected option by transgender participants was that it should also be *removed* from the ICD-11 completely (42.9%; *n* = 222). Thirty-three point six percent (*n* = 174) of TG participants thought the children’s diagnosis should *remain* in the ICD-11. Nearly a quarter of the participants had no opinion (20.1%; *n* = 104) or said it did not matter to them (3.5%; *n* = 18). There were statistically significant differences between the countries, (χ2(3) = 9.51, *p* = .02, Cramer’s *V* = .14) (see Table 2). Among healthcare providers, there was mainly support for the retention of the GIC diagnosis (55.4%; *n* = 56), while 27.7% (*n* = 28) of the HCPs wanted to remove GIC from the ICD-11, 12.9% (*n* = 13) had no opinion and 4.0% (*n* = 4) said it did not matter to them.

**Table 2:** Frequency Table (and Percentage of Column) of Responses of Transgender Participants to the Question: How would you respond if the children’s diagnosis for gender incongruence were to be taken out of the chapter on “Psychiatric disorders”? *

Possible response	Country of data collection		Total (*n* = 518)
	NL (*n* = 199)	UK (*n* = 319)	
It should be removed from the ICD	79 (39.7%)	143 (44.8%)	222 (42.9%)
It should remain in the ICD	66 (33.2%)	108 (33.9%)	174 (33.6%)
No opinion	41 (20.6%)	63 (19.7%)	104 (20.1%)
Doesn't matter	13 (6.5%)	5 (1.6%)	18 (3.5%)

* Responses on this question differed significantly between the countries, χ2(3) = 9.51, *p* = .02, Cramer’s *V* = .14.

Respondents were asked what they thought of the statement that gender incongruence among children is a psychiatric disorder on a seven point scale (from strongly agree (1) to strongly disagree (7)). In the TG group (*n* = 518), the mean (dis)agreement was 5.69 (*SD* = 1.61). There were no differences between the countries (*U* = 34362, *z* = 1.70, *p* = .10, *r* = .07). The mean score of the UK TG participants (*n* = 319) was 5.80 (*SD* = 1.52, mean rank = 267.72) and 5.51 (*SD* = 1.72, mean rank = 246.33) for the NL TG participants (*n* = 199). When the possible responses were grouped into three categories (agree to various degrees, neutral, disagree to various degrees), it was found that 76.1% of the TG respondents (*n* = 394) disagreed (to various degrees) with the statement, 12.4% (*n* = 64) were neutral, and 11.6% (*n* = 60) agreed (to various degrees) with the statement. In line with the results of the transgender participants, the majority of the HCPs (64.4%; *n* = 65) disagreed (to various degrees) with the statement that gender incongruence among children is a psychiatric disorder, while 12.9% (*n* = 13) was neutral and 22.8% (*n* = 23) agreed.

When asked in which chapter participants thought a diagnosis of gender incongruence for children should be included, only 7.2% of the transgender participants said that the GIC diagnosis should not be in the ICD at all (see Table 3). Over one-third of transgender participants (37.5%; *n* = 188) thought the gender incongruence diagnosis for children should be placed in a separate chapter dealing with symptoms/ disorders regarding sexual and gender health, while 26.7% (*n* = 134) preferred to include gender incongruence of childhood as a Z-code (see Table 3 for all responses). The responses differed statistically between the Netherlands and the UK, (χ2(8) = 38.21, *p* < .01, Cramer’s *V* = .28). See Table 3. In the UK, the most frequently selected option of transgender participants was to include a gender incongruence diagnosis as a Z-code (32.6%; *n* = 104) and secondly to place it in a separate chapter dealing with symptoms and/or disorders regarding sexual and gender health (30.4%; *n* = 97). Most NL transgender participants preferred gender incongruence to be placed in a separate chapter dealing with symptoms and/or disorders regarding sexual and gender health (50.0%; *n* = 91) and their next preferred placement was as a Z-code (16.5%; *n* = 30). In line with the findings for the transgender participants, healthcare providers (40.0%, *n* = 40) most frequently selected placement in a separate chapter dealing with symptoms and/or disorders regarding sexual and gender health and 24.0% (*n* = 24) preferred to include GIC as a Z-code, while 6.0% (*n* = 6) thought it should not be in the ICD at all.

**Table 3:** Frequency Table (and Percentage of Column) of Responses of Transgender Participants to the Question: In what chapter of the ICD-11 do you think the diagnosis of gender incongruence for children should be included? *

Possible response	Country of data collection		
	NL (*n* = 182)	UK (*n* = 319)	Total (*n* = 501)
Neurologic disorders and diseases	8 (4.4%)	13 (4.1%)	21 (4.2%)
Hormonal disorders and diseases	8 (4.4%)	18 (5.6%)	26 (5.2%)
Urogenital disorders and diseases	0 (0.0%)	2 (0.6%)	2 (0.4%)
Psychiatric disorders and diseases	11 (6.0%)	5 (1.6%)	16 (3.2%)
It should be part of several medical chapters simultaneously	12 (6.6%)	44 (13.8%)	56 (11.2%)
A separate chapter dealing with symptoms / disorders regarding sexual and gender health	91 (50.0%)	97 (30.4%)	188 (37.5%)
It should be a Z-code	30 (16.5%)	104 (32.6%)	134 (26.7%)
It should not be in the ICD at all	12 (6.6%)	24 (7.5%)	36 (7.2%)
Other, namely	10 (5.5%)	12 (3.8%)	22 (4.4%)

* Responses on this question differed significantly between the countries, χ2(8) = 38.21, *p* < .01, Cramer’s *V* = .28.

### Stigmatization due to a diagnosis

Just over a third (36.6%; *n* = 188) of transgender participants had no opinion regarding the question: ‘*Do you think that the proposed diagnosis for children will have a greater stigmatizing effect (i*.*e*. *more so than for adults)*?’ (see Table 4). Just over a third (34.5%; *n* = 177) did not think that the proposed diagnosis for children would have a greater stigmatizing effect for children than for adults. There was a statistically significant difference between the two countries, (χ2(3) = 27.91, *p* < .01, Cramer’s *V* = .23), see Table 4. Some participants elaborated on this answer. For example, a Dutch parent who thought the diagnosis did not have a greater stigmatizing effect for children than for adults wrote: “*When you know something is wrong but cannot figure out what it is*, *a diagnosis is a liberation*. *It is a definition for the problems you are experiencing*. *It can be stigmatizing but that doesn’t mean*, *the diagnosis is bad in itself*”. A parent from the UK wrote: “…*because again*, *society still likes to have a doctor’s opinion on anything*. *So having a diagnosis will make people take you more seriously*. *Problem is until you have that diagnosis*. *Then everyone looks at you like your kid is only having a tantrum over an extra chocolate*.” Examples of parents who thought a diagnosis would have a greater stigmatizing effect for children than for adults include: “*A diagnosis made (too early) will remain in your medical record for ever*. *Even if you are no longer experiencing gender incongruence in puberty*. *It will remain a stigma if we are not careful*.” (Dutch parent) or *“*…*attaching a diagnosis of "incongruence" to a child who is completing a normal process of identity development is premature and implies that the process of identity development is following an "incongruent" path which does not match the norm*. *It is inappropriate to diagnose a condition that will change as the child grows and matures in their sense of self/identity/esteem”* (parent from the UK).

**Table 4:** Frequency Table (and Percentage of Column) of Responses of Transgender Participants to the Question: Do you think that the proposed diagnosis for children will have a greater stigmatising effect (i.e. more so than for adults)? *

Possible response	Country of data collection		Total (*n* = 513)
	NL (*n* = 194)	UK (*n* = 319)	
No	93 (47.9%)	84 (26.3%)	177 (34.5%)
Yes	40 (20.6%)	82 (25.7%)	122 (23.8%)
No opinion	50 (25.8%)	138 (43.3%)	188 (36.6%)
Other, namely…	11 (5.7%)	15 (4.7%)	26 (5.1%)

* Responses on this question differed significantly between the countries, χ2(3) = 27.91, *p* < .01, Cramer’s *V* = .23.

Of the HCPs, just over half (52.5%; *n* = 53) did not think that the proposed diagnosis for children would have a greater stigmatizing effect for children than for adults, 26.7% (*n* = 27) thought it would and 14.9% (*n* = 15) had no opinion.

The UK survey included the following question: ‘*Do you think having a psychiatric diagnosis for gender incongruence could have a beneficial effect for children*?’. The most commonly selected response, by a narrow margin, (39.2%; *n* = 125) was that a psychiatric diagnosis for gender incongruence would *not* have a beneficial effect for children. Yet, 32.9% (*n* = 105) of participants thought this could have a beneficial effect. The others had no opinion (15.4%; *n* = 49) or selected the option ‘Other (please specify)…’ (12.5%; *n* = 40).

The results from HCPs differed from those of the TG participants; most HCPs (45.8%; *n* = 11) thought that a psychiatric diagnosis for gender incongruence could have a beneficial effect for children and 25.0% (*n* = 6) thought it could not.

When asked: ‘*Do you think that a child with gender-incongruent feelings needs gender identity care*?*’*, 78.0% (*n* = 386) of TG participants agreed with this statement (see Table 5). The responses differed statistically between the NL and the UK participants, (χ2(2) = 13.99, *p* < .01, Cramer’s *V* = .17), see Table 5. In line with the findings for the transgender participants, most healthcare providers (58.9%, *n* = 53) thought that a child with gender-incongruent feelings needs gender identity care, or were not sure (34.4%, *n* = 31) while only 6.7% (*n* = 6) did not think so.

**Table 5:** Frequency Table (and Percentage of Column) of Responses of Transgender Participants to the Question: Do you think that a child with gender-incongruent feelings needs gender identity care? *

Possible response	Country of data collection		Total (*n* = 495)
	NL (*n* = 176)	UK (*n* = 319)	
No	4 (2.3%)	7 (2.2%)	11 (2.2%)
Yes	153 (86.9%)	233 (73.0%)	386 (78.0%)
I'm not sure, because…	19 (10.8%)	79 (24.8%)	98 (19.8%)

* Responses on this question differed significantly between the countries, χ2(2) = 13.99, *p* < .01, Cramer’s *V* = .17.

### Stricter criteria of the GIC diagnosis

Responses were mixed regarding the following question: ‘*Do you consider it an improvement that the proposed ICD 11 criteria for the children’s diagnosis will be stricter than in the ICD-10 (children will have to meet all criteria for a period of two years)*?*’*. Slightly more TG participants (38.2%; *n* = 189) answered “no” compared to “yes” (33.5%; *n* = 166) (see Table 6 for all responses). There were statistically significant differences between UK and NL participants, (χ2(3) = 105.37, *p* < .001, Cramer’s *V* = .46). In the UK, the majority response (35.8%) was that this was an improvement, while in the NL the majority response (49.5%) was that this was not an improvement, see Table 6.

**Table 6:** Frequency Table (and Percentage of Column) of Responses of Transgender Participants to the Question: Do you consider it an improvement that the proposed ICD 11 criteria for the children’s diagnosis will be stricter than in the ICD 10 (children will have to meet all criteria for a period of two years)? *

Possible Response	Country of data collection		Total (*n* = 495)
	NL (*n* = 176)	UK (*n* = 319)	
No	31 (17.6%)	158 (49.5%)	189 (38.2%)
Yes	63 (35.8%)	103 (32.3%)	166 (33.5%)
I'm not sure	54 (30.7%)	7 (2.2%)	61 (12.3%)
No opinion	28 (15.9%)	51 (16.0%)	79 (16.0%)

* Responses on this question differed significantly between the countries, χ2(3) = 105.37, *p* < .001, Cramer’s *V* = .46.

Of the HCPs, 46.2% (*n* = 43) thought the stricter criteria for the diagnosis were an improvement; 18.3% (*n* = 17) thought it was not; 20.4% (*n* = 19) was unsure; and 15.1% (*n* = 14) had no opinion.

### Removal distress and impairment criterion

Most TG participants (combining response options 3 and 4: 60.2%; *n* = 298) thought it was desirable if the distress criterion was removed from the children’s diagnosis (see Table 7 for all responses). Most NL (58.5%; *n* = 103) and most UK participants (50.2%; *n* = 160) selected the option: “Desirable, because for both age groups psychological distress should not be a criterion for getting a diagnosis.” The response options differed between the countries, so we cannot make any further comparisons. In line with the findings for the transgender participants, most healthcare providers (49.5%, *n* = 45) thought the removal was desirable.

**Table 7:** Frequency Table (and Percentage of Column) of Responses of Transgender Participants to the Question: How do you feel about removing the distress criterion from the children’s diagnosis (when you think about the children’s diagnosis and the diagnosis for adults)?

Possible Response	Country of data collection		Total (*n* = 495)
	NL (*n* = 176)	UK (*n* = 319)	
Undesirable, because I consider it important for children and adults that they meet the criterion	20 (11.4%)	33 (10.3%)	53 (10.7%)
Undesirable, because I consider it more important for children that they meet the criterion than for adults	10 (5.7%)	25 (7.8%)	35 (7.1%)
Desirable, because I consider it less important for children that they meet the criterion than for adults	11 (6.3%)	24 (7.5%)	35 (7.1%)
Desirable, because for both age groups psychological distress should not be a criterion for getting a diagnosis	103 (58.5%)	160 (50.2%)	263 (53.1%)
No opinion	28 (15.9%)	53 (16.6%)	81 (16.4%)
Other, namely (NL only)	4 (2.3%)	-	-
Undesirable, because. (UK only)	-	16 (5.0%)	-
Desirable, because… (UK only)	-	8 (2.5%)	-

### Results from the healthcare provider only questions

There were more HCPs who thought they would able to take care of children (reimbursed by health care insurance) if there was no children’s diagnosis (*n* = 22, 26.8%), (taking the two ‘yes’ categories together) than HCPs who thought they would not be able to take care of children (*n* = 11, 13.4%) (see Table 8). Leaving out the HCPs to whom the question was not applicable (who did not see children), 51.2% of the participants thought they could provide care for children (the two ‘yes’ categories together) and 25.6% thought they could not. The responses to this question did not differ statistically between the countries (only people to whom the question was applicable were included).

**Table 8:** Frequency Table (and Percentage of Column) of Responses of Health Care Providers to the Question: If no children’s diagnosis existed, would you still be able to treat (and keep treating) children who have gender incongruence (reimbursed by health care insurance)? *

Possible response	Country of data collection		Total (*n* = 82)
	NL (*n* = 61)	UK (*n* = 21)	
Not applicable; I do not assess or treat children	27 (44.3%)	12 (57.1%)	39 (47.6%)
No	6 (9.8%)	5 (23.8%)	11 (13.4%)
Yes, no problem	9 (14.8%)	2 (9.5%)	11 (13.4%)
Yes, but only if I can make another concurrent diagnosis for it (e.g. depression)	10 (16.4%)	1 (4.8%)	11 (13.4%)
Don’t know	9 (14.8%)	1 (4.8%)	10 (12.2%)

* Responses on this question did not differ significantly between the countries, Fisher’s exact test, *p* > .05.

Half of the HCPs (50.0%; *n* = 37) thought the criteria for children were easy to use in their clinic; 5.4% (*n* = 4) disagreed; and 44.6% (*n* = 33) had no opinion. There were no statistically significant differences between the countries. Furthermore, regarding the duration criterion of 2 years, 39.2% (*n* = 29) of the HCPs thought it was *not* difficult to determine whether this criterion was fulfilled; 27.0% (*n* = 20) thought it was difficult to determine; another 27.0% (*n* = 20) had no opinion; and the others (6.8%; *n* = 5) had another response. There were no statistically significant differences between the countries. The highest percentage of HCPs (28.8%; *n* = 21) thought they could properly make the distinction between slight gender variance and a situation in which the criteria for the GIC diagnosis have been met; 19.2% (*n* = 14) thought this would not be easy; 24.7% (*n* = 18) had no opinion; and 27.4% (*n* = 20) did not know. No differences were found between the countries. Of the Dutch HCPs, 35.8% (*n* = 19) thought it would be harder to give a diagnosis if children did not have to express GI feelings than if it were required to express their feelings; 18.9% (*n* = 10) thought it would be easier; 26.4% (*n* = 14) said it did not matter; and 18.9% (*n* = 10) had no opinion (NL only question).

## Discussion

This field trial was set up to receive input on WHO’s proposed ICD-11 criteria for Gender Incongruence of Childhood. A large number of participants were recruited from the Netherlands and the United Kingdom and stakeholders from various backgrounds were represented in the sample, including transgender participants, parents of children with gender incongruent feelings, healthcare providers (some specialized in transgender health care, others who were not), and individuals who fit more than one category. The main aim of this study was to identify whether the GIC diagnosis should be included in the ICD-11. Two questions were asked regarding the placement of GIC in ICD-11. When asked: How would you respond if the children’s diagnosis for gender incongruence (gender dysphoria/gender identity disorder) would be taken out of the chapter on “psychiatric disorders”?, the most frequently cited response favoured removing the diagnosis. Healthcare providers were mostly in favour of retaining the GIC diagnosis within the ICD-11, although about a quarter of them preferred to remove it in its entirety. This study found more support of healthcare providers for retaining the GIC diagnosis in ICD-11, compared to earlier surveys amongst WPATH members (all specialized in transgender healthcare) where no consensus was reached [6,11], although it should be noted that the samples differed as our sample consisted of healthcare providers both specialized and not-specialized in transgender healthcare. Most participants from the UK thought that a psychiatric diagnosis for gender incongruence could *not* have a beneficial effect for children and a smaller, but substantial, group disagreed.

The second question was: “In what part of the ICD-11 do you think the diagnosis of gender incongruence (gender dysphoria/gender identity disorder) for children should be included?”. In response to this question, most transgender participants chose an option within the ICD-11, rather than selecting the response option “It should not be in the ICD at all”. Given the findings above, this appears contradictory, however, this is likely to reflect the participant’s interpretation of the question. I.e. a participant may favour removal, but if that is not possible, where would be the next most appropriate place to move it? Similar findings are described regarding the GI diagnosis in Adolescence/Adulthood [17]. In both cases, the “removal” response option was the eighth option out of nine and the question does not necessarily imply that there would be a response option for *not* being included in the ICD, so some people may have chosen a category that they found acceptable and may not have finished reading all of the options. In other words, their responses might have been based on the idea that *if* the diagnosis is retained, where they thought the best placement option would be. The number of people in favour of removing the GIC diagnosis thus might be higher than the percentage of participants that picked “It should not be in the ICD at all” (7.2%) (see also [17]). Respondents may have also selected the option of leaving the diagnosis within the ICD as they may see having a diagnosis as the only option for accessing clinical services. On the other hand, participants may initially have favoured removal of the diagnosis (first question), but—after seeing the various placement options in the second question—they may have considered the removal unnecessary and concluded that one of the chapters was an appropriate place for the GIC diagnosis.

Participants were generally satisfied with other aspects of the proposed ICD-11 GIC diagnosis. Most transgender participants and stakeholders thought the term *Gender Identity Disorder* should change, and most thought *Gender Incongruence* was an improvement. Furthermore, most participants reported that they did not consider *Gender Incongruence* to be a psychiatric disorder or condition and therefore placement in a separate chapter dealing with Gender and Sexual Health (the majority response in the Netherlands and in the TG group) or as a Z-code (the majority response in the UK) would be preferable. It should be noted that the name of the new chapter was slightly different in the survey (Gender and Sexual Health) than in the current beta draft (Sexual Health) [12]. Similar placement preferences were found regarding the Gender Incongruence of Adolescence and Adulthood (GIAA) diagnosis [17]. The differences between the countries can be at least partially explained by the differences in healthcare systems described in the introduction of this paper. Including GIC as a Z-code might jeopardize access to care for Dutch transgender youth. Therefore, this option might be less popular in the Netherlands than in the United Kingdom, where a Z-code is preferable and access to care is unaffected by this change in placement. This shows that recognition of the context of existing healthcare systems within which questions are being answered is important.

WGSDSH’s suggestion to narrow the diagnosis for children in order to reduce the number of false positives and stigma [2], was not seen as an improvement by a substantial part of the NL sample (49.5%). However, it was seen as an improvement by 32.3% of the NL sample and by 35.8% of the UK sample (the majority response in the UK). Furthermore, in relation to stigma, most transgender participants did not think it was more stigmatizing for children to receive a GI diagnosis than for adolescents or adults. However, this does not indicate the GIC diagnosis itself is not stigmatizing.

Most participants thought that a child with gender incongruent feelings needs gender identity related care. This finding at first sight speaks against the argument that a diagnosis is not needed because medical treatment for gender incongruence is not available for prepubertal children (e.g., [3,6]). It is likely that support and psychological input is seen by participants as useful for children with GIC and their families. Whether or not this type of psychological care is reimbursed, differs across countries and healthcare systems.

Although generally, the results from healthcare providers were in line with the results from the transgender participants (and stakeholders) some differences were found. While most healthcare providers from the UK thought having a psychiatric diagnosis for gender incongruence could have a beneficial effect for children, most transgender participants from the UK disagreed. Interpretation is difficult since the sample size of the healthcare providers from the UK was small. Another difference between the participant groups was that most HCPs thought the stricter GIC criteria to be an improvement, while most TG participants did not. It is interesting that the TG participants have this opinion, as stricter criteria imply that fewer children with gender incongruence will have a diagnosis and that fewer children would experience the potential stigmatizing effects of having a diagnosis. Yet TG participants did not support the stricter criteria and seemed to favour a diagnosis that would include more rather than fewer children. It may well be that these participants, like many in other quarters, struggle with the balance between avoiding stigma and access to appropriate care.

Specific questions for healthcare providers (specialized and not-specialized in transgender related care) showed that they were mainly positive about the proposed children’s diagnosis. Overall, healthcare providers thought: 1) the GIC criteria were easy to use in their clinic; 2) it was *not* difficult to determine whether the duration criterion of experiencing two years of gender incongruence was fulfilled; and 3) the distinction between slight gender variance and a situation in which the criteria for the diagnosis have been met could be determined properly for children. One aspect of the proposed GIC diagnosis was seen as more challenging to determine compared to the ICD-10 criteria: most Dutch HCPs thought the fact that children no longer have to express or verbalize GI feelings in the proposed ICD-11 made the criteria more difficult to use than if it were required that they have done so.

Most HCPs thought they could continue providing care for children if there was no GIC diagnosis. This suggests that most HCPs are not worried about losing access to care for children with GI feelings as a result of there not being a diagnosis in ICD-11. However, some HCPs noted that they could continue to help children only if they could make a concurrent diagnosis (e.g., depression or anxiety). This suggests that some youth who function well and do not have any (mental) health issues might not be able to gain access to care, at least with these health care professionals. One possibility to ensure access to care is for HCPs to use deliberate misdiagnosis (for example, to give a child with gender incongruent feelings a diagnosis of anxiety). This practice of deliberate misdiagnosis is quite common amongst mental health professionals [18,19]. Although mental health providers usually do this with the client’s best interest in mind (e.g., for problems with reimbursement for services if a certain code is used; or to avoid stigmatizing the patient), clearly this is not ideal and may lead to inaccurate information on prevalence, policy and program making [18,19], not to mention the ethical issues involved.

The different views between HCP and transgender participants regarding the need to keep a GIC diagnosis is likely to be related to the variations in the meaning that a “diagnosis” has for different people. Classification systems and diagnoses were developed as a means of ordering information, grouping phenomena and providing a language by which to communicate with other clinicians, researchers and patients and their families. It is widely agreed that diagnostic criteria should, whenever possible, be based on aetiology. However this is not always possible. For a researcher a diagnosis might be useful in order to study different phenomena. Without it, it can make research more complicated, but not impossible. Because of this, clinical academics may value retaining some kind of diagnosis as part of the classification system. Transgender people may view things differently. For many years, the psychopathologisation of transgender people has been linked to its categorization as a psychiatric diagnosis. Transgender people have suffered for many years the discrimination and stigmatisation of being considered psychiatric patients by clinicians and wider society. Unsurprisingly for transgender people a diagnosis may always be linked to stigma and removing it all together makes sense. They may argue that if gay and lesbian people are no longer considered part of a classification system, why then should transgender people be. For parents of young people with gender incongruence a diagnosis may be linked to support and advice. Many parents with a child with gender incongruence may feel isolated and lost. For those parents having somewhere to attend in order to discuss their distress and concerns is vital. If health services are organised in a way that a diagnosis is necessary for access, for parents a diagnosis will be linked to support and help. Parents may suggest keeping a diagnosis in order to have access to services, but is this the right reason to keep a diagnosis? This survey did not include one of the most difficult questions to answer: what does a diagnosis mean to you? Future researchers may want to consider this, as it may help to interpret their findings.

### Limitations

Next to the limitations mentioned above regarding the possible influence of the order of response options, wording of the questions and the low response rate. There are some additional points to consider also. First, the majority of the transgender participants were individuals who intended to receive, were receiving, or had received gender identity care (see S3 Text). This group may be less opposed to GI diagnoses in ICD-11 since they receive(d) reimbursed medical treatment made possible (in the NL at least) through a diagnostic code. In other words, people who want to receive, have received, or are receiving medical care are benefitting from the presence of a diagnosis because they are eligible for reimbursed health care. As a result, they might be more positive and less worried about the possible stigmatizing effect of including GI diagnoses in ICD-11 when compared to transgender people who do not use medical interventions or who have used them a long time ago. It is therefore important to realize that this study’s sample is not representative of all transgender people in the Netherlands and the UK (see also [17]).

A second limitation was that the survey was long and involved some complex concepts which may have made it difficult to understand for some participants. Indeed, the sample was biased towards participants with a high level of education. Also, the length of the survey may have discouraged participants from completing it (see also [17]). Ideally, we would have included the opinions of the children themselves, but the issues are too complex and abstract for them to understand, particularly in the context of a long questionnaire. Rather, face-to-face interviews would have been preferable with young people, but the time and costs associated with this were prohibitive.

A third limitation specific to this study regarding the *Gender Incongruence of Childhood* diagnosis is that we do not know if the adult transgender participants had received a gender incongruence diagnosis in childhood and have had first-hand experiences with the care provided and possible negative (e.g. stigmatization) or positive (e.g., access to care and information) outcomes of receiving a diagnosis. Such experiences would clearly be important in shaping opinions regarding the diagnosis.

### Conclusion

In conclusion, this study suggests that although in an ideal world a diagnosis relating to gender incongruence in children is not welcomed, several participants felt the diagnosis should not be removed. This is likely due to concerns about restricting access to reimbursed healthcare. Furthermore, this study found that the majority choice for positioning of a diagnosis of *Gender Incongruence* within the ICD-11 is as a separate chapter dealing with conditions regarding sexual and gender health. This was the overall first choice for participants in The Netherlands and second choice for those in the UK, behind the use of a Z-code. The difference reflects the fact that in the UK, Z-codes carry no negative implications of non-reimbursement of treatment costs. This clearly demonstrates the added layer of complexity that comes with having to consider financial implications and not just psychological, social, and moral ones. These findings highlight the challenges faced by the WHO in their attempt to integrate research findings from different countries, with different cultures and healthcare systems and to create a manual that is globally applicable and usable.

## Supporting Information

S1 Text The UK Survey (this article reports on the questions marked in yellow). (DOCX)

S2 Text The ICD-11 draft criteria of ‘Gender Incongruence of Childhood’ of the WGSDSH criteria used in this study. (DOCX)

S3 Text Additional information on the background of the respondents. (DOCX)
