# Supplementary material for: Gender Incongruence of Childhood: Clinical Utility and Stakeholder Agreement with the World Health Organization’s Proposed ICD-11 Criteria
Source: PLoS One. 2017 Jan 12;12(1):e0168522. doi: 10.1371/journal.pone.0168522 (PMC5233419; doi:10.1371/journal.pone.0168522)
Supplement: S1 Text — (DOCX) [file pone.0168522.s001.docx]

**S1 Text. The UK Survey (this article reports on the questions marked in yellow)**

**Opinions regarding the proposed ICD-11 criteria of the WHO**

***Disclaimer***

***Please note that this is an international survey translated from an original Dutch version and consequently some terms may seem unfamiliar. All effort has been made, together with the trans communities, to make this as understandable and inclusive as possible and so for the purpose of this survey the word trans covers all trans identities.Nonetheless please accept our apologies for anything that you may find offensive. All feedback is welcome.***

***Disclaimer***

**Introduction**

The World Health Organization (WHO) uses a classification system for all diseases and disorders for which medical or psychological care may be required. Hence, this also applies to psychiatric diseases and disorders. The system is called the *International Classification of Diseases* (ICD). It is used for collecting all sorts of statistical details regarding a country’s healthcare system and for putting in place the appropriate policies. The WHO is currently engaged in preparing a new version of this system, the ICD-11 (see the Supplement at the end of this survey for the proposed criteria).

You may know that, at the moment, a discussion is being held about the diagnoses included in the current version, ICD-10. In this version, the gender identity diagnosis for adults is called *transsexuality*, and that for children is called *gender identity disorder*.

The WHO would like to incorporate in this discussion not only the opinions of specialists in the field, but also those of non-specialist caregivers, the transgender persons themselves, and the parents/carers of young transgender persons.

We would therefore like to know your opinions about the main issues.

In order to know what various groups (caregivers, transgender persons, parents/carers) think about various issues, we will also ask a number of questions about yourself.

Since the proposed name of the diagnosis is *gender incongruence*, we will use this name instead of *gender dysphoria*.

**Some questions about yourself**

The following questions allow us to describe the demographics (age, education, geographical location, etc) of the people that have completed the survey. This is important in order to ensure that no groups are under-represented.

**Please note**: If you are completing this questionnaire on behalf of a young person, please answer the following questions about them and not yourself.

1. Where do you live?

- England
- Scotland
- Wales
- Northern Ireland
- Other (please specify) ........................................................................................

1. In which part of the country do you live? (optional)

| - Aberdeenshire | - East Lothian | - Orkney |
| --- | --- | --- |
| - Anglesey | - Essex | - Oxfordshire |
| - Angus | - Fermanagh | - Peeblesshire |
| - Antrim | - Fife | - Pembrokeshire |
| - Argyll | - Flintshire | - Perthshire |
| - Armagh | - Glamorgan | - Radnorshire |
| - Ayrshire | - Gloucestershire | - Renfrewshire |
| - Banffshire | - Hampshire | - Ross-shire |
| - Bedfordshire | - Herefordshire | - Roxburghshire |
| - Berkshire | - Hertfordshire | - Rutland |
| - Berwickshire | - Huntingdonshire | - Selkirkshire |
| - Brecknockshire | - Inverness-shire | - Shetland |
| - Buckinghamshire | - Kent | - Shropshire |
| - Buteshire | - Kincardineshire | - Somerset |
| - Caernarfonshire | - Kinross-shire | - Staffordshire |
| - Caithness | - Kirkcudbrightshire | - Stirlingshire |
| - Cambridgeshire | - Lanarkshire | - Suffolk |
| - Cardiganshire | - Lancashire | - Surrey |
| - Carmarthenshire | - Leicestershire | - Sussex |
| - Cheshire | - Lincolnshire | - Sutherland |
| - Clackmannanshire | - Londonderry | - Tyrone |
| - Cornwall | - Merionethshire | - Warwickshire |
| - Cromartyshire | - Middlesex | - West Lothian |
| - Cumberland | - Midlothian | - Westmorland |
| - Denbighshire | - Monmouthshire | - Wigtownshire |
| - Derbyshire | - Montgomeryshire | - Wiltshire |
| - Devon | - Morayshire | - Worcestershire |
| - Dorset | - Nairnshire | - Yorkshire |
| - Down | - Norfolk | - **Prefer not to say** |
| - Dumbartonshire | - Northamptonshire | - **Other**   **(please specify)**  **.......................................** |
| - Dumfriesshire | - Northumberland |  |
| - Durham | - Nottinghamshire |  |

1. How would you describe your gender identity?
   - I identify as a man (including with a trans history)
   - I identify as a woman (including with a trans history)
   - I identify partly as a man, and partly as a woman
   - I identify neither as a man, nor as a woman
   - I don’t know what my gender identity is (yet) or I am questioning my gender identity
   - Other (please specify) ……………………..…….........................................................
2. What gender was assigned to you at birth?

- Male
- Female
- Neither, as I have the following condition/ type of diversity(please specify) .......................................................................................................................................

........................................................................................................................................

1. How do you identify yourself?

- Trans*
- Trans
- Transvestite
- Transgender
- Transsexual
- Cisgender (you are content to remain the gender you were assigned at birth)
- Non-binary gender
- Gender neutral / neutrois
- Pangender
- Bigender
- Genderqueer
- Androgynous
- Other (please specify) ..............................................................................................

1. What is your age? ...................................
2. In what year did you start living permanently in your desired gender role? ...................
3. Do you feel that there should be a diagnosis related to being trans?

- Yes
- No
- No opinion

Please explain your answer.................................................................................................

..........................................................................................................................................................................................................................................................................................................................................................................................................................................

1. Do you feel that the current diagnosis includes non-binary people sufficiently?

- Yes
- No
- No opinion

Please explain your answer ..........................................................................................

........................................................................................................................................

1. What is your highest level of education?

- Pre-GSCE / CSE, Standard Grades
- Secondary Education (e.g., GCSE / CSE, Standard Grades)
- Upper Secondary/Further Education (e.g., A/S Level, A-Level/O-Level/Highers, NVQs, BTEC, HND)
- Bachelor’s degree
- Master’s Degree / MPhil
- Doctorate Degree / PhD
- Other (please specify) ...............…………………….....................................

1. Are you...? *Please tick all that apply*

- A psychological or medical practitioner specialised in transgender care inside a specialised gender centre
- A psychological or medical practitioner specialised in transgender care outside a specialised gender centre
- A psychological or medical practitioner not specialised in transgender care
- A teacher (primary/secondary school / further education)
- A trans person who intends to receive, who is receiving, or has received gender identity care
- A trans person who is not receiving transgender care, nor intends to receive it
- A trans person’s parent/guardian
- A trans person’s sibling
- A trans person’s child
- A trans person’s partner
- A professional who works in an advocacy/support capacity regarding gender identity care
- An academic researcher
- Other (please specify).....................................................................................................

The following section contains questions for **clinicians** and **trans individuals**

- If you are a ***clinician***, please answer **Questions 11a, 11b, 11c & 11d** (marked (C))
- If you are a ***trans person***, please answer **Question 11d, 11m, 11n & 11o** (marked (T))
- If you are a ***clinician and a trans person***, please **continue and answer all** of the questions.
- If you are a ***family member of a trans person***, please move on to **Question 12.**

11a. (C) At what organisation do you work? (optional) ………………………………………………………………………………………………………………………………………………………………………………………………………………

Specify your responsibility/position:……………………………………………………………………………………………………………………………………………………………………………………………………………..

11b. (C)Do you do diagnostic work in the field of gender incongruence? (previously known as gender dysphoria/gender identity disorder)

- No
- Yes

11c. (C)Do you give treatment in the field of gender incongruence?(previously known as gender dysphoria/gender identity disorder)

- No
- Yes, I provide psychosocial / psychotherapeutic/ psychological / psychiatric care
- Yes, I provide medical care (hormones)
- Yes, I provide medical care (surgical procedures)
- Yes, other (please specify)..............................................................................

11d.(C & T) Do you have contact with other trans people? *Please tick all that apply*

- No
- In the past, but not now
- Yes, they are my clients/patients
- Yes, via the Internet
- Yes, I interact socially with some trans people
- Yes, I am active in a trans organisation

11m. (T) Are you undergoing hormone treatment as part of your gender identity care (e.g. oestrogens, testosterone, or puberty delaying hormone)?

- Not applicable
- No and I never have
- No, but I have previously (please specify).........................................................
- Not yet, but I would like to
- Yes I am taking the following hormone treatment(s)…………...........................
- No and I never have

11n.(T) Have you had any surgical procedures as part of your gender identity care?

- Not applicable
- No and I do not intend to
- No, I am undecided/considering it
- Not yet, but I would like to
- Yes, I have had the following procedures……………………….........................

11o.(T) If you wish to have further surgeries, which would you like? ……………........................................................................................................................................................................................................................................................................................................................................................................................................................

**Question 12. Questions about your opinion on the proposed diagnostic criteria**

The following set of questions will ask for your opinions are on the current and the proposed diagnostic criteria related to what is currently called transsexualism.

These questions will ask your opinions on the terminology used, where it is positioned in the diagnostic manual, and what you feel would be the impact of changing this (for example in terms of stigma, treatment pathways, diagnosis, etc).

The questions will consider children (aged 11 and under), adolescents (aged 12 - 15), and adults (aged 16+) separately.

**General features of the diagnosis**

*Terminology*

1. Which of the following options best describes your opinion of the statement:

“The diagnostic term for gender incongruence **for adolescents and adults** now used in the ICD-10 is *transsexuality*. The term *transsexuality* must change.”

- Strongly agree
- Agree
- Agree a little
- Neutral; neither agree, nor disagree
- Disagree a little
- Disagree
- Strongly disagree

Please explain your answer.......................................................................................................

……………………………………………………………………………………………………………………………………………………………………………………………………............................

1. Which of the following options best describes your opinion of the statement:

“The diagnostic term for gender incongruence **for children** now used in the ICD-10 is *gender identity disorder.* This term must change.”

- Strongly agree
- Agree
- Agree a little
- Neutral; neither agree, nor disagree
- Disagree a little
- Disagree
- Strongly disagree

Please explain your answer…….................................................................................................

........................................................................................................................................................................................................................................................................................................

1. Do you consider the proposed name of *gender incongruence* an improvement on *transsexualism?*

- No
- Yes
- It doesn’t really matter to me what term is used
- I have no opinion on this

Please explain your answer……................................................................................................

......................................................................................................................................................................................................................................................................................................

1. Do you consider the proposed name of *gender incongruence* an improvement on *gender identity disorder*?

- No
- Yes
- It doesn’t really matter to me what term is used
- I have no opinion on this

Please explain your answer……...............................................................................................

..................................................................................................................................................

..................................................................................................................................................

1. What diagnostic term would you prefer using yourself?

- Gender transition
- Gender diversity
- Gender variance
- Gender incongruence
- Gender dysphoria
- Trans
- Transsexuality
- Gender identity disorder
- Other (please specify)...............................................................................................

**NOTE**: **Questions 17 a, b, c & d** is for **clinicians only.**

If you are **not**a clinician, please move on to **Question 18 .**

17a. (C) Do your patients,clients or their parents /guardians ever make any negative comments about the term *transsexualism?*

- Not applicable
- No
- Yes, sometimes
- Yes, often

If yes, please can you give a briefexample…………………………………………………………

…………………………………………………………………………………………………………..

…………………………………………………………………………………………………………..

17b.(C) Do your patients, clients or their parents/guardians ever make any negative comments about the term *gender identity disorder*?

- Not applicable
- No
- Yes, sometimes
- Yes, often

If yes, please can you give a brief example………………………………………………………….

……………………………………………………………………………………………………………

……………………………………………………………………………………………………………

17c. (C) Do your colleagues ever make any negative comments about the term *transsexualism*?

- Not applicable
- No
- Yes, sometimes
- Yes, often

If yes, please can you give a brief example …………………………………………………………

……………………………………………………………………………………………………………

……………………………………………………………………………………………………………

17d. (C) Do your colleagues ever make any negative comments about the term *gender identity disorder*?

- Not applicable
- No
- Yes, sometimes
- Yes, often

If yes, please can you give a brief example…………………………………………………………

………………………………………………………………………………………………………………………………………………………………………………………………………………………..

**Question 18.** The following section is for **ALL** respondents.

*Positioning of the diagnosis* **among adolescents/adults**

1. Which of the following options best describes your opinion of the statement:

“Gender incongruence (gender dysphoria/gender identity disorder) in and of itself **among adolescents/adults** is a psychiatric disorder”.

- Strongly agree
- Agree
- Agree a little
- Neutral; neither agree, nor disagree
- Disagree a little
- Disagree
- Strongly disagree

1. If you *do not* consider gender incongruence(gender dysphoria/gender identity disorder) **among adolescents/adults** a psychiatric disorder, what would you say it is? ………………………………………………………………………………………………………..
2. How would you respond if the **adolescents/adults** diagnosis for gender incongruence (gender dysphoria/gender identity disorder) were to be taken out of the chapter on “psychiatric disorders”?

- It should be completely removed from the ICD
- It should be allowed to remain in the ICD
- No opinion
- Doesn’t matter

1. Do you think having a psychiatric diagnosis for gender incongruence (gender dysphoria/gender identity disorder)has a stigmatising effect for **adults and adolescents**?

- No
- Yes
- No opinion
- Other (please specify)....................................................................................................

1. Do you think having a psychiatric diagnosis for gender incongruence (gender dysphoria/gender identity disorder) could have a beneficial effect for **adults and adolescents?**

- No
- Yes
- No opinion
- Other (please specify)...................................................................................................

*Positioning of the diagnosis* **among children**

1. Which of the following options best describes your opinion of the statement:

“Gender incongruence **among children** is a psychiatric disorder”.

- Strongly agree very much
- Agree
- Agree a little
- Neutral; neither agree, nor disagree
- Disagree a little
- Disagree
- Strongly disagree very much

1. If you do not consider gender incongruence **among children** a psychiatric disorder, what would you say it is? ……………………………………………………………………………………………………………………………………………………………………………………………………………….
2. How would you respond if the children’s diagnosis for gender incongruence (gender dysphoria/gender identity disorder) would be taken out of the chapter on “psychiatric disorders”?

- It should be completely removed from the ICD
- It should be allowed to remain in the ICD
- No opinion
- Doesn’t matter

1. Do you think that the proposed diagnosis for **children** will have a greater

stigmatising effect (i.e. *more so than for adults*)?

- No, because (please specify) ........................................................................................................................................ ........................…............................................................................................................
- Yes, because (please specify)

...............................................................................................................................................................................................................................................................................

- No opinion
- Other(please specify) .......................................................................................................................................

.......................................................................................................................................

1. Do you think having a psychiatric diagnosis for gender incongruence (gender dysphoria/gender identity disorder) could have a beneficial effect for **children**?

- No
- Yes
- No opinion
- Other (please specify) ....................................................................................................

1. Do you think having any kind of psychiatric diagnosis has a stigmatising effect? By this we mean general psychiatric diagnoses, rather than specific gender diagnoses.

- No
- Yes
- No opinion
- Other (please specify) ....................................................................................................

*Positioning of the diagnosis – General*

**NOTE**: **Questions 29 a and b** are for **trans individuals only.**

If you are **not** a trans individual, please move on to **Question 30.**

29a. (T) Have you ever been discriminated against for any of the following reasons? Please tick all that apply.

- Gender-variant behaviour
- Gender-dysphoric feelings
- The way in which you express your gender identity by your dress or hair style (your gender expression)
- Your gender diagnosis
- I have never been discriminated against
- Other reason (please specify)......................................................................................

If yes, please could you give a brief example?……………………………………………………

…………………………………………………………………………………………………………..

29b. (T) Have you experienced any benefits or positives from being a trans person?

- Yes
- No

Please explain your answer………………………………............................................................

...................................................................................................................................................

*………………………………………………………………………………………………………………………………………………………*

**Question 30.** The following section is for **ALL** respondents.

30. Do you see any other negative consequences of having a diagnosis for gender incongruence?

- Yes
- No

Please explain your answer……………………………….............................................................

....................................................................................................................................................

1. Do you see any other positive consequences of having a diagnosis for gender incongruence?

- Yes
- No

Please explain your answer………………………………...........................................................

..................................................................................................................................................

1. Do you think having **any kind** psychiatric diagnosis (including diagnoses other than gender diagnoses) affords validation of your identity, practice or issues?

- No
- Yes
- No opinion
- Other (please specify) .................................................................................................

Please explain your answer……………………………….........................................................

.................................................................................................................................................

1. Do you think having a diagnosis for **gender incongruence** (gender dysphoria/gender identity disorder) affords validation of your identity, practice or issues?

- No
- Yes
- No opinion
- Other (please specify) ....................................................................................................

Please explain your answer………………………………............................................................

....................................................................................................................................................

1. In what chapter of the ICD-11 do you think the diagnosis of gender incongruence(gender dysphoria/gender identity disorder) **for adolescents/adults** should be included?

- Neurologic disorders and diseases
- Hormonal disorders and diseases
- Urogenital disorders and diseases
- Psychiatric disorders and diseases
- It should be part of several medical chapters simultaneously
- A separate chapter dealing with symptoms / disorders regarding sexual and gender health (*Conditions Related to Sexual and Gender Health)*
- It should be a Z-code (this concerns “factors that affect health and also influence contacts with the healthcare system” but does not concern diseases or disorders)
- It should not be in the ICD at all
- Other (please specify) …...............................................................................................

Please explain your answer.......................................................................................................

...................................................................................................................................................

1. In what part of the ICD-11 do you think the diagnosis of gender incongruence (gender dysphoria/gender identity disorder) **for children** should be included?

- Neurologic disorders and diseases
- Hormonal disorders and diseases
- Urogenital disorders and diseases
- Psychiatric disorders and diseases
- It should be part of several medical chapters simultaneously
- A separate chapter dealing with symptoms / disorders regarding sexual and gender health (*Conditions Related to Sexual and Gender Health)*
- It should be a Z-code (this concerns “factors that affect health and also influence contacts with the healthcare system” but does not concern diseases or disorders)
- It should not be in the ICD at all
- Other (please specify) …............................................................................................

Please explain your answer....................................................................................................

................................................................................................................................................

**The diagnosis for adolescents and adults**

*Widening the scope of the diagnosis*

The proposed diagnosis for **adolescents and adults** has now been formulated in such a way that it also includes other forms of gender incongruence apart from the traditional category of *transsexuality*. Hence the scope of the diagnosis has been widened.

**NOTE: Questions 36 a and b**are for **clinicians only.**

If you **are not** a clinician, please move on to **Question 37.**

36a.Have you already met with clients/patients in your work to whom this wider scope of diagnosis might apply (for instance, persons who do not feel themselves to be either man or woman, and wish to live their lives as gender-neutral, non-binary persons)?

- Not applicable; I don’t work in the (mental) healthcare sector
- No, never
- Yes, sometimes
- Yes, regularly
- Other (please specifiy)..................................................................................................

36b. Do you think that you yourself will receive more requests for treatment if the scope of the diagnosis is widened?

- Not applicable; I don’t work in the (mental) healthcare sector
- I don’t know
- I think so, but I’m not sure
- Definitely

**Question 37.** The following section is for **ALL** respondents.

37. Do you think that, if the scope of the diagnosis is widened, more people who do not experience themselves to be either man or woman or who experience themselves to be outside the box of manhood or womanhood, etc. will seek treatment?

- I don’t know
- I don’t think so
- I think so, but I’m not sure
- Definitely

38. Would you consider it an improvement if the scope of the diagnosis were widened? (several answers may apply)

- No, the risk is too high that people will get the diagnosis even though they don’t need any help. I think it’s stigmatising.
- No, because (please specify)........................................................................................

......................................................................................................................................

- Yes, but there is a danger that too many people will get the diagnosis; even those who do not need it. That is stigmatising.
- Yes, treatment will thus become available to a more diverse and larger group
- Yes, because (please specify).......................................................................................

........................................................................................................................................

- No opinion

39. If you had to choose between 1) the risk of *too many* people getting a diagnosis and 2) the risk of *too few* people getting a diagnosis, what would you choose?

- 1) that *too many* people get a diagnosis
- 2) that *too few* people get a diagnosis
- I don’t know

If you would like to expand further on this, please explain your choice .............................

............................................................................................................................................

40. Do you expect that there will be changes in healthcare funding for a gender diagnosis if the diagnosis changes?

- No
- No opinion
- Yes (please specify)……………………………..............................................................

......................................................................................................................................

*Leaving out the criterion of distress and impairment*

In the proposed diagnosis, it will no longer be required for a person who has gender incongruence to experience *serious distress* or *functional impairment* (e.g. inability to function properly in their job or social life) in order to get the diagnosis. Formerly, people would *only* be eligible for the diagnosis if he/she experienced serious distress from the gender incongruence or was unable to function properly because of it. So it was only then that they could get treatment covered by providers. After all, without a diagnosis, no treatment is required.

41. The **proposed change** means that people who experience discomfort with their gender-assignment at birth **but who do not** experience significant distress that impairs their ability to live a functional life (work, socialise, etc) will now meet the diagnostic criteria regardless.

Do you think that would be an improvement? (several answers may apply)

- No, because, this way, the diagnosis will also apply to people who experience gender incongruence but don’t have a problem with it. This stigmatises a large group of people.
- No, because, without experiencing distress, a person should not be able to get a diagnosis.
- No, because (please specify) .............................................................................

............................................................................................................................

- Yes, because, this way, people who do *not experience distress* from their gender incongruence but who *do* wish to receive treatment will be able to get a diagnosis and become eligible for treatment.
- Yes, because leaving out the criterion of psychological distress means that the diagnosis can be taken out of the psychiatric chapter.
- Yes, because (please specify)........................................................................... …………………………………………………………………………………………..
- No opinion
- Doesn’t matter
- Other…................................................................................................................

*Duration of gender incongruence*

In the proposed diagnosis for **adolescents and adults**,it will be a requirement for gender incongruence (gender dysphoria/gender identity disorder) to have lasted for *a few months*. This is to prevent people with highly fluctuating or very recent gender incongruence from getting a premature diagnosis. In the previous ICD version, a period of two years applied.

1. How do you respond to limiting the duration of gender incongruence to a few months?

- Too short
- Still too long
- No opinion
- Other

Please explain your answer……………………………………............................................

1. What time period would you suggest is appropriate?

- 0-6 months
- Minimum of 1 year
- Minimum of 2 years
- Minimum of 3 years
- Minimum of 4 years
- 5 + years

Please explain your answer...............................................................................................

*DSD-specification*

1. Do you think that persons with physical sex development disorders (also known as intersex disorders or *Disorders/Differences of Sex Development* (DSD)) and who may experience gender incongruence later on as a result of an incorrect gender assignment at birth should be able to get the diagnosis?

- No
- Yes
- No opinion
- Doesn’t matter
- Other

Please explain your answer……………………….......................................................................

The following question is for **clinicians only.**

If you are **not** a clinician please move on to **Question 46.**

**The children’s diagnosis**

*Consequences of the children’s diagnosis*

45a. (C) If no children’s diagnosis existed, would you still be able to treat (and keep treating) children who have gender incongruence(gender dysphoria/gender identity disorder)?

- Not applicable; I do not assess or treat children
- No
- Yes
- Yes, but only if I can come up with some other diagnosis for it (e.g. depression)
- Don’t know

**Question 46**. The following section is for **ALL** respondents.

*Criteria of the children’s diagnosis*

In the current ICD 10, the diagnosis criteria for children states that Gender Identity disorder of childhood is:

“A disorder, usually first manifest during early childhood (and always well before puberty), characterized by a persistent and intense distress about assigned sex, together with a desire to be (or insistence that one is) of the other sex. There is a persistent preoccupation with the dress and activities of the opposite sex and repudiation of the individual's own sex. The diagnosis requires a profound disturbance of the normal gender identity; mere tomboyishness in girls or girlish behaviour in boys is not sufficient.”

1. The **proposed ICD 11 criteria for children**’s diagnosis will be stricter, in that children will have to meet all criteria for a period of two years. Do you consider this an improvement?

- No
- Yes
- No opinion

Please explain your answer .....................................................................................

*Removing the criterion of distress and impairment*

1. As in the diagnosis for adolescents and adults, in the proposed **children’s** diagnosis, it will no longer be a prerequisite for the child to experience serious distress from gender incongruence in order to meet the criteria.

Do you think that would be an improvement? (several answers may apply)

- No, because this way the diagnosis will also apply to children who experience gender incongruence but don’t have a problem with it. This stigmatises a large group of children.
- No, because, without experiencing distress, a person should not be able to get a diagnosis; if serious distress is one of the criteria, this will undermine the arguments for removing the diagnosis from the psychiatric chapter.
- No, because (please specify).......................................................................................

......................................................................................................................................

- Yes, because, this way, children who do *not experience distress* from their gender incongruence but who *do* wish to receive care will be able to get a diagnosis and become eligible for care (which will be covered by the NHS).
- Yes, because leaving out the criterion of psychological distress means that the diagnosis can be removed from the psychiatric chapter.
- Yes, because (please specify).......................................................................................

.......................................................................................................................................

- No opinion
- Doesn’t matter
- Other…..................................................................................................................................................................................................................................................................

1. With regard to the proposed children’s diagnosis, it is no longer a prerequisite that the child has to experience serious distress from gender incongruence in order to meet the criteria.

How do you feel about that?

- Undesirable, because I consider it important for both children and adults that they meet the criterion for psychological distress
- Undesirable, because I consider it more important for children that they meet the criterion for psychological distress than for adults
- Undesirable, because………………………………………………………………...............

.......................................................................................................................................

- Desirable, because I consider it less important for children that they meet the criterion for psychological distress than for adults
- Desirable, because, for both age groups, psychological distress should not be a criterion for getting a diagnosis
- Desirable, because………………………………………………………..............................

.......................................................................................................................................

- No opinion

Do you think that a child with gender-incongruent feelings needs gender identity care and treatment etc?

- No
- Yes
- I’m not sure

Please explain your answer......................................................................................

1. Perhaps not all the aspects that you consider relevant have been raised in this questionnaire, or perhaps your answers require some extra clarification. Below you can specify your additional ideas or clarify your answers.

NOTE: If you do give a clarification/comment to a specific question, please state the number of the question you are responding to.

**NOTE:The following section is for clinicians only.**

If you are**not**a clinician, please go to **Question 52**.

Use of the diagnosis

The following questions concern the clarity and applicability of the proposed ICD-11 diagnosis for **adolescents/adults**and for **children**.

*Clarity*

51a. Do you think the diagnosis has been written down *clearly*?

- No
- Yes
- No opinion

51.a. If you think the diagnosis is unclear, does this apply to:

i. The criteria for adolescents/adults

- No
- Yes, because….............................................................................
- No opinion

ii.The criteria for children

- No
- Yes, because ….......................................................................
- No opinion

51.a.iii. How do you feel about the diagnostic criteria for adults?

- The wording is too general
- The wording is too specific
- The wording is appropriate and useful
- No opinion
- Other

Please explain your answer................................................................................

51.a.iv.How do you feel about the diagnostic criteria for children?

- The wording is too general
- The wording is too specific
- The wording is appropriate and useful
- No opinion
- Other

Please explain your answer....................................................................................

*Applicability*

51.b. Do you think the diagnostic criteria for **adolescents / adults** *are easy to use* in your practice/work?

- No
- Yes
- No opinion

Please explain your answer...........................................................................................

.......................................................................................................................................

51.c. Do you think the diagnostic criteria for **children** *are easy to use* in your practice/work?

- No
- Yes
- No opinion

Please explain your answer........................................................................................

....................................................................................................................................

51.d. One of the indicators of gender incongruence among children is:

“A strong desire on the child’s part to be a different gender than the assigned sex, or insistence that he or she is a gender different from one’s assigned gender.”

The child does not necessarily have to express these feelings through their gender role. This is to prevent the possibility that a child who lives in an oppressive environment is unable to get the diagnosis by virtue of the fact that theydo not dare to express their feelings through their gender role.

As a result of this, do you think that the diagnosis:

- Is easier to make than if it were required that his/her feelings are expressed through their gender role
- Is harder to make than if it were required that his/her feelings are expressed through their gender role
- Doesn’t matter
- No opinion

51.e. Do you think in the required period (a few months) during which **adolescents/ adults** have to experience gender incongruence, the diagnosis is:

- Not difficult to determine
- Difficult to determine
- No opinion
- Other

Please explain your answer................................................................................

51.f. Do you think in the required period (two years) during which **children** have to experience gender incongruence, the diagnosis:

- Not difficult to determine
- Difficult to determine
- No opinion
- Other

Please explain your answer......................................................................................

51.g. Do you think the distinction between slight gender variance and a situation in which the criteria for the diagnosis have been met can be determined properly **for adolescents/adults**?

- No
- Yes
- No opinion
- Don’t know

Please explain your answer......................................................................................

51.h. Do you think the distinction between minor variations from the norm and a situation in which the criteria for the diagnosis have been met can be determined properly **for children**?

- No
- Yes
- No opinion
- Don’t know

Please explain your answer ......................................................................................

51.i.Does the fact that serious distress or functional impairment is not a mandatory criterion make it harder for you to make the diagnosis?

- No
- Yes
- No opinion
- Don’t know

Please explain your answer........................................................................................

**Question 52**. The following section is for **ALL** respondents.

**Opinions about the position of transgender persons in healthcare and ideas in society about transgender persons**

*Position of transgender persons in healthcare*

The following questions concern ***your opinion.***

52. Please state your opinion of the following statement:

“Nobody but the transgender persons themselves can determine or diagnose their gender incongruence. Psychologists or psychiaters should not get involved in this”.

- - Strongly agree
  - Agree
  - Neither agree, nor disagree
  - Disagree
  - Strongly disagree

Please explain your answer........................................................................................

**Regarding adults**

53. Please state your opinion of the following statement:

“Decisions on *hormone treatment* should only be taken by the trans adult themselves and the therapist only plays an advisory role.”

- - Strongly agree
  - Agree
  - Neither agree, nor disagree
  - Disagree
  - Strongly disagree

Please explain your answer .........................................................................................

54. Please state your opinion of the following statement:

“Decisions on *surgical procedures*should only be taken by the *trans adult* themselvesand the therapist only plays an advisory role.”

- - Strongly agree
  - Agree
  - Neither agree, nor disagree
  - Disagree
  - Strongly disagree

Please explain your answer ..........................................................................................

**Regarding children**

55.Please state your opinion of the following statement:

“Who should make the decision about *hormone* treatment for children(< 12 years old)?”

- - The child, and the therapist and parents/guardians only play an advisory role.
    - Strongly agree
    - Agree
    - Neither agree, nor disagree
    - Disagree
    - Strongly disagree

Please explain your answer.................................................................

56.Please state your opinion of the following statement:

“Who should make the decision about *hormone* treatment for children(< 12 years old)?”

- - The parents/guardians, in consultation with the child, and the therapist only plays an advisory role.
    - Strongly agree
    - Agree
    - Neither agree, nor disagree
    - Disagree
    - Strongly disagree

Please explain your answer.................................................................

1. Please state your opinion of the following statement:

“Children (<12 years old) should be allowed to decide about hormone treatment and surgical procedures, even if their parents/guardians do not agree with their choice.”

- - Strongly agree
  - Agree
  - Neither agree, nor disagree
  - Disagree
  - Strongly disagree

Please explain your answer..........................................................................

1. Please state your opinion of the following statement:

“For children under the age of 12, an **official solicitor** should protect the child’s rights if parents/guardians and child do not agree with each other.”

- - Strongly agree
  - Agree
  - Neither agree, nor disagree
  - Disagree
  - Strongly disagree

Please explain your answer........................................................................................

1. Please state your opinion of the following statement:

“Decisions about *surgical procedures* should be postponed until the child reaches an age where they are legally allowed to decide for themselves (at the age of 16).”

- - Strongly agree
  - Agree
  - Neither agree, nor disagree
  - Disagree
  - Strongly disagree

Please explain your answer......................................................................................

**Regarding adolescents**

1. Please state your opinion of the following question:

“Who should make the decision about ***hormone***treatment for adolescents(12-15 years old)?”

- - The adolescent, and the therapist and parents/guardians only play an advisory role.
    - Strongly agree
    - Agree
    - Neither agree, nor disagree
    - Disagree
    - Strongly disagree

Please explain your answer....................................................................

1. Please state your opinion of the following question:

“Who should make the decision about ***hormone***treatment for adolescents(12-15 years old)?”

- - The parents/guardians, in consultation with the adolescent, and the therapist only plays an advisory role.
    - Strongly agree
    - Agree
    - Neither agree, nor disagree
    - Disagree
    - Strongly disagree

Please explain your answer.....................................................................

1. Please state your opinion of the following question:

“Who should make the decision about ***surgical*** proceduresfor adolescents(12-15 years old)?”

- - The adolescent, and the therapist and parents/guardians only play an advisory role.
    - Strongly agree
    - Agree
    - Neither agree, nor disagree
    - Disagree
    - Strongly disagree

Please explain your answer.................................................................

1. Please state your opinion of the following question:

“Who should make the decision about ***surgical*** proceduresfor adolescents(12-15 years old)?”

- - The parents/guardians, in consultation with the adolescent, and the therapist only plays an advisory role.
- Strongly agree
- Agree
- Neither agree, nor disagree
- Disagree
- Strongly disagree

Please explain your answer...................................................................

1. Adolescents (12-15 years old) should be allowed to make decisions about hormone treatment and surgical procedures, even if their parents/guardians do not agree with their choice.
   - Strongly agree
   - Agree
   - Neither agree, nor disagree
   - Disagree
   - Strongly disagree

Please explain your answer................................................................................

1. Currently, the right of trans people to take decisions regarding their choice of medical treatment is sufficiently respected.
   - Strongly agree
   - Agree
   - Neither agree, nor disagree
   - Disagree
   - Strongly disagree

Please explain your answer.................................................................................

**Your ideas about gender in society.**

Please indicate how much you agree with the following statements:

1. People can belong to only one of two genders – male or female
   - Strongly agree
   - Agree
   - Neither agree, nor disagree
   - Disagree
   - Strongly disagree

Please explain your answer......................................................................................................

1. It is important that children are raised either wholly as a boy or wholly as a girl.
   - Strongly agree
   - Agree
   - Neither agree, nor disagree
   - Disagree
   - Strongly disagree

Please explain your answer...................................................................................................

1. It is important to all trans people to use hormone treatment and surgical procedures in order to go through life as ordinary men and women.
   - Strongly agree
   - Agree
   - Neither agree, nor disagree
   - Disagree
   - Strongly disagree

Please explain your answer ....................................................................................................

1. Trans feelings are accepted in society.
   - Strongly agree
   - Agree
   - Neither agree, nor disagree
   - Disagree
   - Strongly disagree

Please explain your answer.................................................................................................

1. Trans people are widely accepted in society

- Strongly agree
- Agree
- Neither agree, nor disagree
- Disagree
- Strongly disagree

Please explain your answer............................................................................................

**This is the end of the survey**

If you feel that certain things have not been addressed in the survey or that your answers require further explanation, please use the room below to add any additional comments you may have.

…………………………………………………………………………………………………………………………………………………………………………………………………………………………………………………………………………

…………………………………………………………………………………………………………………………………………………………………………………………………………………………………………………………………………

- - Should you have any **further questions** about the survey, please email them to [icd.who@nottshc.nhs.uk](mailto:icd.who@nottshc.nhs.uk)
  - If you would like to be **informed about the results** of this survey, please email or [icd.who@nottshc.nhs.uk](mailto:icd.who@nottshc.nhs.uk) leave your email address here:

……………………………………………………………………………………………………………...............................................................................................................

- - If you would like to **take part in future studies**, please email your name and contact details to [icd.who@nottshc.nhs.uk](mailto:icd.who@nottshc.nhs.uk) or leave your email address here: ……………………………………………………………………………………………………….......................................................................................................................

**We thank you for taking part in this study.**

**Your time and support is appreciated.**

**Supplement 1. Proposed ICD-11 criteria for children**

**Definition:**

Gender Incongruence of Childhood is characterized by a marked incongruence between an individual’s experienced/expressed gender and the assigned sex in pre-pubertal children.

**Essential (Required) Features:**

- In pre-pubertal children, a marked incongruence between the child’s experienced/expressed gender and the child’s assigned sex as manifested by all of the following indicators:
- A strong desire on the child’s part to be a different gender than the assigned sex, or insistence that he or she is a gender different from one’s assigned gender.
- A strong dislike on the child’s part of his or her sexual anatomy or anticipated secondary sex characteristics and/or a strong desire for the primary and/or anticipated secondary sex characteristics that match the experienced gender. For example, a child assigned at birth as a boy says he wants to be rid of his penis or a child assigned at birth as a girl says she does not want to develop breasts when she grows up.
- Make-believe or fantasy play, toys, games, or activities and playmates that are typical of their experienced rather than their assigned sex. Gender incongruent children assigned as boys reject typically “masculine” toys, games, and activities and avoid rough-and-tumble play. Gender incongruent children assigned as girls reject “feminine” toys, games, and activities and like rough-and tumble play.
- The incongruence must have persisted for about 2 years.Although some indications of Gender Incongruence may be present when children are as young as age 2, it is not possible to perform an accurate assessment of Gender Incongruence of Childhood at this age.The requirement of a duration of about 2 years implies that the diagnosis cannot be made before approximately age 5.
- The diagnosis can only be assigned to children before puberty.

**Associated Clinical Presentations:**

- Gender Incongruence of Childhood may be associated with clinically significant distress or impairment in social, school, or other important areas of functioning, particularly in disapproving social environments, but neither distress nor functional impairment is a diagnostic requirement.
- Children with Gender Incongruence of Childhood are at increased risk of poor peer relations, social isolation, and poor academic performance because of negative social attitudes and stigmatization.
- Gender incongruent children in disapproving social environments are at increased risk of psychological distress, psychiatric symptoms and physical injuries related to social rejection and exclusion, bullying, and violence.
- Clinically referred children diagnosed with Gender Incongruence of Childhood who have co-occurring mental and behavioral disorders exhibit a predominance of “internalizing” as compared to “externalizing” disorders.
- Common co-occurring conditions among children with Gender Incongruence of Childhood include anxiety disorders.
- Autism spectrum disorders are more prevalent among clinically referred children with Gender Incongruence of Childhood than among the general population

**Supplement 2. Proposed ICD-11 criteria for adolescents/adults**

**Definition:**

Gender Incongruence of Adolescence and Adulthood is characterized by a marked and persistent incongruence between an individual´s experienced gender and the assigned sex, which often leads to a desire to ‘transition’, in order to live and be accepted as a person of the experienced gender, through hormonal treatment, surgery or other health care services to make the individual´s body align, as much as desired and to the extent possible, with the experienced gender. The diagnosis cannot be assigned prior to the onset of puberty.

**Essential (Required) Features:**

- In adolescents and adults, a marked incongruence between the individual´s experienced gender and the assigned sex, as manifested by at least two of the following:
  - A strong dislike or discomfort with the one´s primary and/or secondary sex characteristics (in adolescents, anticipated secondary sex characteristics) due to their incongruity with the experienced gender.
  - A strong desire to be rid of some or all or one´s primary and/or secondary sex characteristics (in adolescents, anticipated secondary sex characteristics) due to their incongruity with the experienced gender.
  - A strong desire to have the primary and/or secondary sex characteristics of the experienced gender.
  - A strong desire to be treated (live and be accepted) as a person of the experienced gender.
- The experienced gender incongruence must have been continuously present for at least several months.
- The diagnosis cannot be assigned prior to the onset of puberty.

**Associated Clinical Presentations:**

- Gender Incongruence of Adolescence and Adulthood may be associated with clinically significant distress or impairment in social, occupational, or other important areas of functioning, particularly in disapproving social environments, but neither distress nor functional impairment is a diagnostic requirement.
- An inability to live as the experienced gender may be associated with impairment in social, occupational, or other important areas of functioning, particularly in disapproving social environments where protective laws and policies are absent, often as a result of stigmatization and social exclusion.
- Individuals with Gender Incongruence are at increased risk of social isolation, school drop-out, loss of employment, homelessness, and disrupted interpersonal relationships because of negative social attitudes and stigmatization.
- Gender incongruent adolescents and adults are at increased risk of psychological distress, psychiatric symptoms and physical injuries related to social rejection, stigmatization, victimization, and violence.
- Common co-occurring conditions include depressive and anxiety disorders. In adolescents, the onset of puberty and the impending development of secondary sex characteristics incongruent with the experienced gender may lead to severe anxiety states and suicidal ideation.
- Autism spectrum disorders are more prevalent among clinically referred adolescents and adults with Gender Incongruence than among the general population.

**Supplement 3. ICD-10 Gender identity disorder of childhood**

Disorders, usually first manifest during early childhood (and always well before puberty), characterized by a persistent and intense distress about assigned sex, together with a desire to be (or insistence that one is) of the other sex. There is a persistent preoccupation with the dress and/or activities of the opposite sex and/or repudiation of the patient's own sex.

These disorders are thought to be relatively uncommon and should not be confused with the much more frequent nonconformity with stereotypic sex role behaviour. The diagnosis of gender identify disorder in childhood requires a profound disturbance of the normal sense of maleness or femaleness; mere 'tomboyishness' in girls or 'girlish' behaviour in boys is not sufficient. The diagnosis cannot be made when the individual has reached puberty.

**Supplement 4. ICD-10 Transsexualism**

A desire to live and be accepted as a member of the opposite sex, usually accompanied by a sense of discomfort with, or inappropriateness of, one's anatomic sex and a wish to have hormonal treatment and surgery to make one's body as congruent as possible with the preferred sex.
