# Supplementary material for: Gender Incongruence of Childhood: Clinical Utility and Stakeholder Agreement with the World Health Organization’s Proposed ICD-11 Criteria
Source: PLoS One. 2017 Jan 12;12(1):e0168522. doi: 10.1371/journal.pone.0168522 (PMC5233419; doi:10.1371/journal.pone.0168522)
Supplement: S2 Text — (DOCX) [file pone.0168522.s002.docx]

**S2 Text. The ICD-11 draft criteria of ‘Gender Incongruence of Childhood of the WGSDSH criteria used in this study.**

**Definition:**

Gender Incongruence of Childhood is characterized by a marked incongruence between an individual’s experienced/expressed gender and the assigned sex in pre-pubertal children.

**Essential (Required) Features:**

- In pre-pubertal children, a marked incongruence between the child’s experienced/expressed gender and the child’s assigned sex as manifested by all of the following indicators:
- A strong desire on the child’s part to be a different gender than the assigned sex, or insistence that he or she is a gender different from the assigned gender.
- A strong dislike on the child’s part of his or her sexual anatomy or anticipated secondary sex characteristics and/or a strong desire for the primary and/or anticipated secondary sex characteristics that match the experienced gender. For example, a child assigned at birth as a boy says he wants to be rid of his penis or a child assigned at birth as a girl says she does not want to develop breasts when she grows up.
- Make-believe or fantasy play, toys, games, or activities and playmates that are typical of their experienced rather than their assigned sex. Gender incongruent children assigned as boys reject typically “masculine” toys, games, and activities and avoid rough-and-tumble play. Gender incongruent children assigned as girls reject “feminine” toys, games, and activities and like rough-and tumble play.
- The incongruence must have persisted for about 2 years. Although some indications of Gender Incongruence may be present when children are as young as age 2, it is not possible to perform an accurate assessment of Gender Incongruence of Childhood at this age. The requirement of a duration of about 2 years implies that the diagnosis cannot be made before approximately age 5.
- The diagnosis can only be assigned to children before puberty.
